# An Empirical Analysis of Overlap Publication in Chinese Language and English Research Manuscripts

**DOI:** 10.1371/journal.pone.0022149

**Published:** 2011-07-12

**Authors:** Joseph D. Tucker, Helena Chang, Allison Brandt, Xing Gao, Margaret Lin, Jing Luo, Philip Song, Kai Sun, Xiaoxi Zhang

**Affiliations:** 1 Department of Medicine, Massachusetts General Hospital, Boston, Massachusetts, United States of America; 2 Department of Medicine, University of North Carolina, Chapel Hill, North Carolina, United States of America; 3 Department of Medicine, University of Wisconsin, Madison, Wisconsin, United States of America; 4 Department of International Development, Oxford University, Oxford, United Kingdom; 5 United States Centers for Disease Control, Beijing, China; 6 Department of Medicine, Washington University, St. Louis, Missouri, United States of America; 7 Department of Medicine, University of Illinois at Chicago, Chicago, Illinois, United States of America; 8 Department of Medicine, Johns Hopkins University, Baltimore, Maryland, United States of America; 9 Department of Medicine, University College London, United Kingdom; IUMSP, CHUV/University of Lausanne, Switzerland

## Abstract

**Background:**

There are a number of sound justifications for publishing nearly identical information in Chinese and English medical journals, assuming several conditions are met. Although overlap publication is perceived as undesirable and ethically questionable in Europe and North America, it may serve an important function in some regions where English is not the native tongue. There is no empirical data on the nature and degree of overlap publication in English and Chinese language journals.

**Methods/Principal Findings:**

A random sample of 100 English manuscripts from Chinese institutions was selected from PubMed. Key words and institutions were searched in the China National Knowledge Infrastructure, a comprehensive Chinese language research database. Unacknowledged overlap was *a priori* defined according to International Committee of Medical Journal Editor (ICMJE) guidelines following examination by two individuals. 19% (95% CI 11–27) of English manuscripts from Chinese institutions were found to have substantial overlap with Chinese published work based on full text examination. None of the manuscripts met all of the criteria established by the ICMJE for an acknowledged overlap publication. Individual-level, journal-level, and institutional factors seem to influence overlap publication. Manuscripts associated with an institution outside of China and with more than one institution were significantly less likely to have substantial overlap (p<0.05).

**Conclusions/Significance:**

Overlap publication was common in this context, but instances of standard ICMJE notations to acknowledge this practice were rare. This research did not cite the identified overlap manuscripts with the hope that these empirical data will inform journal policy changes and structural initiatives to promote clearer policies and manuscripts.

## Introduction

China is rapidly becoming a scientific powerhouse, fuelled by substantial government investment and support. Tracking along with the growth of scientific innovation in China, publishing in English journals has become an essential part of scientific career advancement. In 2009 Chinese researchers produced over 120,000 manuscripts, second only to the United States [1]. Since there are few highly cited Chinese journals and most scientists outside China do not speak Chinese, one important way for Chinese language manuscripts to reach broader audiences is through translation and re-publication in English. The International Committee of Medical Journal Editors (ICMJE) guidelines provide a clear standard for acknowledging overlap publication in two languages. Yet there is substantial heterogeneity in Chinese journal guidelines, ranging from general approval for any overlap publication [2], [3], [4], [5] to much more detailed guidelines consistent with ICMJE recommendations [6], [7], [8].

Previous empirical work in this field has been heavily influenced by moral trappings and focused on punitive measures. One study went as far as to call duplicate publication a “major sin of modern publishing” [9]. Many discussions of overlap publication have focused on ICMJE recommendations and global standards that should be recognized in all research settings [9], [10]. While recommendations and guidelines are essential to clarify standards, the complex context of publishing the same research in two languages has not been fully explored. Instead of considering overlap publication as individual errors associated with single scientists, this investigation considers overlap publication using a systems framework.

The systems approach has been widely applied to understand medical errors such as wrong-limb amputation or medication overdoses. Through this approach, these errors can be viewed as the end product of a series of problems in the healthcare system, rather than the egregious negligence of individuals [11]. While individual responsibility remains an integral part of quality healthcare, the systems approach acknowledges that individuals are prone to make errors and systems modifications can reduce the prevalence of such errors.

The application of the systems framework to the publication process requires the assumption that an unacknowledged overlap publication is approached as an error rather than a malicious act. While there are many situations in which an author's actions can have ethical or moral implications, the systems framework allows for an objective appraisal of the entire situation and can offer more comprehensive solutions than punitive measures alone. Seen in this perspective, unacknowledged overlap publication can be thought of as more than the simple intent of individual authors to bolster their curriculum vitae or the oversight of a single journal editor or reviewer. The broader context of individual, institutional, and journal factors that promote or discourage unacknowledged overlap publication has important implications.

Aspects of a system that lends itself to errors include “difficulties in information access, tolerance of stylistic practices, and fear of punishment that inhibits reporting” [12]. Other contributing factors can include “national culture, organizational culture, professional culture…[and] vague policies” [13]. These factors are beyond the control of individuals, constitute latent systemic faults that can lead to delayed effects, and are the root causes of errors [14]. Within this system, multiple faults must align for an unacknowledged overlap publication to occur, such as authors not notifying a journal of a preexisting publication, co-authors being unaware that one author is re-submitting an article, editors being unaware of the previous article, and journals with unclear policies. Understood in this context, a systems approach has great potential value in understanding the substance and notation of overlap publications.

Other studies have examined overlap publication in the English scientific literature. An analysis of PubMed abstracts found that 1.35% of entries were highly similar, with research from Japan and China representing roughly twice the duplication rates compared to their relative Medline contributions [9]. One analysis of 1234 publications included in 56 systematic reviews found a prevalence of “covert duplicate publication” of 5.3% [15]. Our study used full text analysis to compare overlap and non-overlap manuscripts among a randomly selected sample of 100 English language manuscripts from Chinese institutions. The purpose of this study was not only to examine overlap publication and its proper notation, but also to situate this scientific discussion within an appropriate systems framework that has the potential to help clarify and broaden the voice of non-English speaking scientific communities.

## Methods

A total of 58,816 PubMed manuscripts were identified using the following search limits: institution in China, time 01/01/00–12/04/09, humans, and English language. Manuscripts that were reviews, case reports, or letters were excluded. 100 manuscripts were randomly selected from this set of PubMed manuscripts using a random number generator. As the primary purpose of this investigation was to investigate the extent of overlap publication, there was not an *a priori* sample size calculation. Each of these English manuscripts was stored in a database with author names, key words, and institutions. Previous literature suggested that authors may not be retained in overlap publications [15], informing our decision to focus search algorithms on key words and institutions from English manuscripts to search in the Chinese National Knowledge Infrastructure (CNKI) database. The CNKI database is a comprehensive searchable Chinese journal database with 8,460 titles since 1994. There were two main reasons why the CNKI database was chosen instead of other databases: 1) CNKI has a free searchable database in which one can view citations and abstracts; 2) Other databases focus on medicine or biological/natural sciences alone while CNKI offers more comprehensive coverage (including subjects such as physics, chemistry, metallurgy, engineering, agriculture). A team of five research assistants fluent in both English and Chinese examined abstracts for potential overlap. All abstracts were examined by a second research assistant before being classified as a possible overlap publication in order to limit type 1 error. Manuscripts with some overlap between the English language version and the Chinese language version were categorized as substantial or minor overlap. Substantial overlap was *a priori* defined as greater than 30% similarity in the content of the introduction, methods, results, or discussion sections. Minor overlap was *a priori* defined as less than 30% similarity in any of the content of these single sections. Classification of similarity within a single article section was based on an individual (not a computer or algorithm) comparing the two manuscript sections.

The systems approach had several implications for this research study: 1) we did not cite or identify any of the overlap manuscripts discovered as part of this research; 2) we examined several journal-level and institutional-level correlates associated with overlap publication; and 3) we used these observational research findings as a basis to consider systems strategies for responding to overlap publication in two languages.

The complete English and Chinese manuscripts of all possible overlap manuscripts were examined in detail. Data from Chinese manuscripts included date of publication, journal name, title or footnote denoting overlap, extent of overlap in the introduction, extent of overlap in the methods, extent of overlap in the results, extent of overlap in the discussion, and number of English and Chinese references. The reference manuscript was pre-specified as the one that was published earlier (regardless of language) and estimations of similarity were made in comparison to the reference. Data from English manuscripts included journal, date of publication, number of institutions represented, co-authors or funding from outside of China, and first author's primary affiliation in Taiwan or Hong Kong. The English journal's 5-year Information Sciences Institute (ISI) impact factor and article influence score were analyzed. We used the article influence score because of the limitations of using impact factor alone [16] and bibliometric research suggesting its utility [17]. The article influence score measures the relative importance of a journal on a per-article basis with the mean score set at 1.0 based on the average article from the Thomson Reuters Journal Citation Reports (JCR) database. Data were coded and Fisher tests were used to analyze associations. Means were compared using a t-test. The main analysis compared the 19 English manuscripts with substantial overlap to the 81 English manuscripts without substantial overlap. 95% confidence intervals were calculated. P-values for t-tests were calculated and reported in the text. All data analysis was done using SPSS 17.0.

## Results

**Figure 1:**
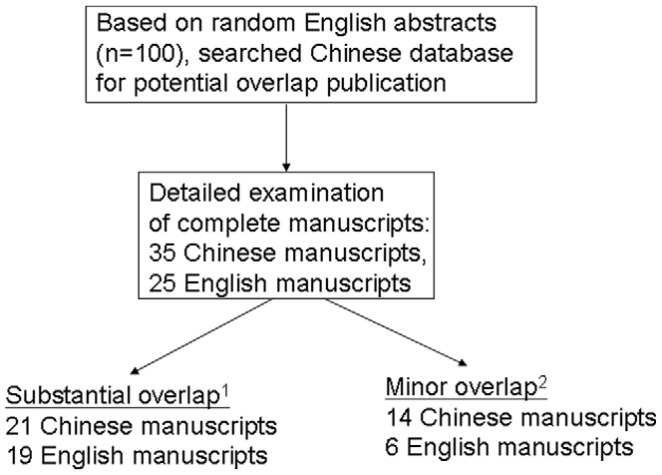
Outline of search strategy for English-language and Chinese-language manuscripts. ^1^Substantial overlap defined by at least 30% similarity in the introduction, methods, results, or discussion. ^2^Minor overlap defined by less than 30% similarity in the introduction, methods, results, and discussion.

This project quantitatively assessed ICMJE compliance and journal and institution-level factors associated with overlap publication. A total of 37 Chinese potential overlap manuscripts were identified by CNKI database searches (Figure 1), and two Chinese full texts could not be found. These 35 potential overlap manuscripts were similar to 25 English manuscripts. There was no difference in the percentage of overlap abstracts identified between different research assistants (p = 0.66). Based on full text examination, 19% (95% CI 11–27) of all English manuscripts had substantial overlap with Chinese published literature and 6% (95% CI 1–11) of all English manuscripts had minor overlap with Chinese published literature. These English manuscripts most often had a single Chinese overlap manuscript (13 single overlaps), but there were five instances of two Chinese manuscripts overlapping with a single English manuscript and four instances of three Chinese manuscripts overlapping with a single English manuscript. 5% (95% CI 1–9) of all English manuscripts were completely identical or nearly identical to published Chinese manuscripts.

Insufficient notation regarding overlap publication according to International Committee of Medical Journal Editor (ICMJE) overlap guidelines was common in this sample. ICMJE guidelines for an acceptable acknowledged overlap publication include the following: 1) title and footnote on the secondary version; 2) separated by one week; 3) secondary version consistent with the primary version; 4) approval from both journal editors; and 5) secondary version intended for a different audience. No manuscripts had a title reflecting previous publication and only one of the English manuscripts had a footnote denoting previous publication. While one of the manuscript pairs was concurrently published, the median time between publication of the two manuscripts was 18.8 months +/−13.7 months. The consistency of the primary and secondary version was quantitatively evaluated with higher numbers associated with greater similarity. The median combined similarity score among overlap papers was 11 out of a maximum score of 16 (interquartile range, 5.5–13.5). Although no communication with journal editors was done as part of this study, analyzing the extent to which the secondary overlap manuscript cited the primary manuscript may serve as a coarse proxy. Among the entire overlap sample (n = 35), only three (8.5%) of the later manuscripts referenced the earlier manuscripts. This study did not assess the audience criteria, but it seems reasonable to assume that Chinese-only speaking and English-only speaking audiences are sufficiently different.

Analyzing medical journal factors illuminated other aspects of overlap publication. Among the 19 manuscripts published in English journals with substantial overlap, 12 had an earlier Chinese version followed by an English version while seven had an earlier English version followed by a Chinese version. Among the 12 English journals that published unacknowledged overlap, all had ethics guidelines for publishing available online and four had explicit overlap guidelines. However, only one journal contained detailed information about overlap publication in more than one language. Most of the Chinese language overlap manuscripts (31/35, 89%) were published in journals that are not indexed in Medline. Abstracts were available for 31 out of the 35 Chinese overlap manuscripts, and among those with abstracts, 90% had English-language titles, author names, keywords, and abstracts in the CNKI database. Manuscripts with substantial overlap were significantly more likely to have a lower ISI impact factor (Table 1).

**Table 1:** Characteristics of substantial overlap manuscripts compared to manuscripts without substantial overlap (n = 100).

	No substantial overlap (n = 81)	Substantial overlap papers (n = 19)	p-value
English journal impact factor mean (SD**)	2.64 (1.99)	1.67 (0.88)	0.009
English journal article influence factor mean (SD)	0.85 (0.84)	0.47 (0.35)	0.06
Mean number of institutions (SD)	2.3 (1.5)	1.5 (0.7)	0.02
Published before 2004 (95% CI)	16.0% (8.8–23.2)	0 (NA)	0.12
More than one institution (95% CI)	65.4% (55.7–74.4)	34.6% (25.7–44.3)	0.04
Institutions outside China* (95% CI)	21.0% (13.0–29.0)	0 (NA)	0.04
Funding outside China* (95% CI)	10.3% (4.1–15.8)	0 (NA)	0.33
First author from Hong Kong or Taiwan (95% CI)	19.8% (12.2–27.8)	5.3% (0.7–9.3)	0.18

*China here includes Hong Kong and Taiwan.

**Standard deviation.

Institutional factors such as location and number of institutions were also analyzed. Not having overlap was associated with having an institution outside of China and having more than one institution represented (Table 1). There was a trend towards greater unacknowledged overlap in later years, but this was not significant (p = 0.12). Other measured variables were not significantly associated with overlap publication.

## Discussion

This study found that 19% of English manuscripts with an author's institution in China had significant overlap after analyzing full texts. The exhaustive manual full text comparison used to analyze similarity made false negatives (type II error) unlikely and the similar overlap detection rates between research assistants further supports the reliability of this approach. This amount of manuscript English/Chinese publication overlap is substantially greater than that found in English systematic reviews (5.3%) [15], PubMed English papers (1.35%) [9], or discipline-specific searches (0.6–14%) [18], [19], [20]. However, one Chinese journal found that 31% of research submissions had some overlap [21]. Since we only searched one of the five major Chinese research databases [22], our estimate for the frequency of unacknowledged overlap publication is likely conservative. This magnitude of unacknowledged overlap publication suggests that there are major structural incentives for publishing the same findings in both English and Chinese.

This study found poor adherence to ICMJE guidelines, and none of the manuscripts contained appropriate notation acknowledging overlap publication. Aside from overlap manuscript pairs being published more than a single week apart and being directed to different audiences, the other major criteria for overlap publishing were nearly universally absent from both Chinese and English manuscripts.

The findings of this empirical analysis suggest the necessity of systems measures to clarify overlap publication at both journal and institutional levels. Among journals, the average impact factor of journals that published overlap publications was lower, consistent with more overlap in journals that are cited less frequently. However, journals with an impact factor as high as 4.1 were also found to have published substantial overlap. A large number of Chinese journals did not have overlap statements that adhere to international consensus statements. The lack of clear online ethics guidelines among Chinese journals limits available data and reveals the potential for authors to overlook such guidelines when they are available. Many Chinese colleges and universities do not have regular access to international journals [23], [24], [25]. Lack of clear overlap journal policies was not restricted to Chinese journals. 12 English manuscripts were published following the original Chinese manuscripts, and inspection of those journal guidelines showed that 75% (8/12) had no specific overlap publication guidelines. The finding that manuscripts representing institutions outside of China had less overlap publication could represent the influence of collaborators outside China to prevent or discourage overlap publication. The trend that having more than one institution was associated with a lower risk of overlap publication supports the notion that isolated research communities have a greater need for research translation and republication.

The findings of this empirical study suggest at least three points where systems interventions could curb unacknowledged overlap publication: 1) clearer journal policies; 2) expanded databases to facilitate broader reviewer searches; and 3) the continued development of Chinese medical research ethics. Clearer journal guidelines on overlap publication are essential for dealing with this issue. One partial solution may be to use checklists as a required part of the manuscript submission process [26]. Journals without such tools can consider their implementation, as they provide a checkpoint in the system that reduces the risk of a notification being forgotten or omitted in a cover letter. Journals with extant requirements can revise their checklists to construct a cogent checkpoint that simultaneously notifies authors of a requirement and receives acknowledgement of that requirement. When dealing with publications that are later discovered to contain unacknowledged overlap, it may be useful to examine non-punitive systems-based reporting programs that have been successful in other medicine contexts [27]. Several Chinese journals have already taken strong positions on the unacceptability of duplicate publication [7], [8], [21], creating momentum for changes in standard editorial processes and expectations. It should also be noted that overlap publications were not only found in Chinese journals, and many English journals also need to clarify and standardize their policy on unacknowledged overlap manuscripts.

Expanded databases to facilitate broader reviewer searches can provide another important checkpoint for evaluating and responding to overlap publication. Only 13% of the Chinese manuscripts found in this analysis were indexed on Medline and thus findable using publicly available similarity analysis tools [28]. At the same time, the majority of the Chinese manuscripts included an English title, authors, abstract, and keywords. This finding underlines the importance of greater cooperation between Chinese databases and PubMed to facilitate searching non-Chinese and non-English manuscripts, respectively. More complete citation listings at PubMed and Chinese databases would help editors and reviewers as they consider original research manuscripts.

The potential contribution of Chinese medical research ethics, as opposed to Euro-American models, in responding to overlap publication should be highlighted. Major Chinese government agencies and research institutions have already adopted many of the formal ethical requirements necessary to undertake US National Institutes of Health research [29]. Therefore, the problem is not that Chinese researchers and government officials underestimate or misunderstand basic principles of Euro-American medical research ethics. Chinese medical research ethics have already made great strides in the last decade [30], and are sufficiently multicultural and inclusive to organize a thoughtful and cogent response to unacknowledged overlap publication [31]. Continuing to develop and nurture medical research ethics in China will contribute to the development of standards and practices that more closely reflect the international consensus on acknowledged overlap publication.

This analysis has several important limitations. First, the search strategy of having two research assistants search each Chinese language abstract minimized false positives (type 1 error), but may have missed less significant overlap. Second, this study relied on subjective analysis of overlap. While subjective measures have been used in other studies assessing overlap [15], quantitative similarity measures are increasingly an option for detection of similar manuscripts [9]. Third, no additional information from authors, journal editors, or institutions were collected as part of this research study, so more detailed analysis involving funding from pharmaceutical companies and other groups was not possible. There have been reports of individual investigators receiving large bonuses for publishing first-author papers in science citation indexed journals [32], but this analysis could not investigate to what extent this motivated unacknowledged overlap publication in China. Finally, this study only analyzed a single Chinese database and so may underestimate the total extent of overlap publication in this specific context.

Although this analysis focused on research manuscripts from Chinese institutions, the findings of this study are relevant to editors, reviewers, and authors in a number of locations. Unacknowledged overlap publication in English and non-English language journals is certainly not confined to Chinese researchers. The high output of Chinese language research manuscripts and the availability of a large public Chinese language database provided a unique opportunity to quantitatively explore the context and notation of overlap publication. Punishment and a moralistic attitude towards unacknowledged overlap publication fail to appreciate the complex journal and institutional factors that have brought about the current situation and which could be targeted to enhance compliance with ICMJE notation. This was a central reason why we decided not to cite the overlap manuscripts. Expanding databases, clarifying journal guidelines, and nurturing the further development of medical research ethics in China are three small steps towards change. There are already indications that top Chinese government officials are concerned about the integrity of published research in Chinese academic centers [33], [34]. Our empirical findings may reflect increasing trends of medical and scientific professionals in China to publish and share their important work according to international publishing standards. As a result of this ongoing professionalization of publication practices, we expect that overlap publication will become less and less common. The data presented provide an opportunity and an appropriate framework to help bring about reform.
